# The windsock deformity complicating endoscopic retrograde cholangiopancreatography planning: an intraluminal duodenal diverticulum

**DOI:** 10.1055/a-2849-6170

**Published:** 2026-04-24

**Authors:** Vicente Lorenzo-Zúñiga

**Affiliations:** 1 Department of GastroenterologyHospital Universitario y Politécnico La Fe. Instituto de Investigación Sanitaria La Fe (IISLaFe)ValenciaSpain; 2 253344 Catholic University of ValenciaValenciaSpain

Intraluminal duodenal diverticulum (IDD), also known as the “windsock” deformity, is a rare congenital anomaly caused by incomplete recanalization of the embryonic duodenum. Progressive elongation of the residual mucosal diaphragm may result in a tubular intraluminal sac that produces a characteristic double-lumen appearance during endoscopy 1 2 3 .

A 49-year-old woman with Down syndrome and symptomatic cholelithiasis was referred for upper gastrointestinal endoscopy because of persistent epigastric pain. Abdominal computed tomography demonstrated gallstones without clear evidence of choledocholithiasis. Because the biliary disease was suspected, endoscopic retrograde cholangiopancreatography (ERCP) was performed.

**Fig. 1:**
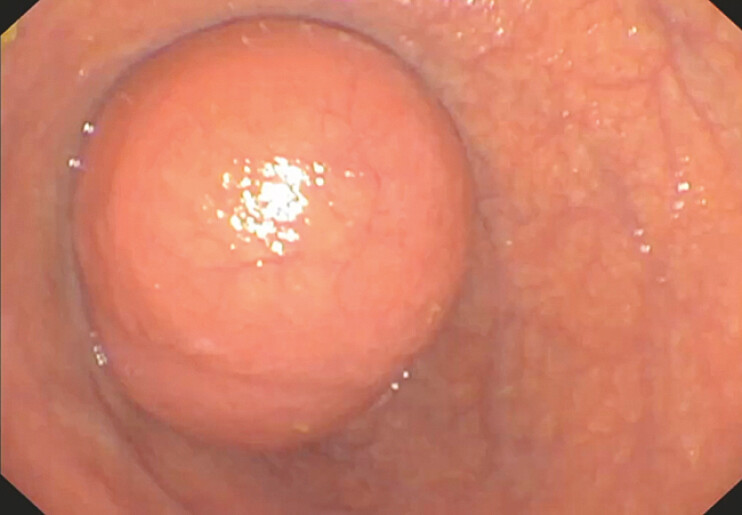
An endoscopic view of a rounded intraluminal sac in the second portion of the duodenum, consistent with an intraluminal duodenal diverticulum (“windsock” deformity).

**Fig. 2:**
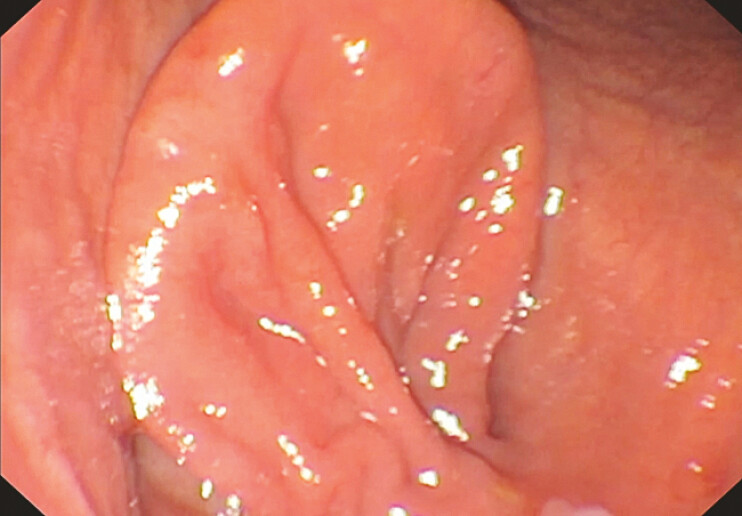
Identification of the true duodenal lumen adjacent to the diverticular sac during careful endoscopic inspection.

Upper endoscopy revealed a rounded intraluminal sac in the second portion of the duodenum covered by normal mucosa, consistent with an intraluminal duodenal diverticulum ( Fig. 1 ; Video 1 ). The lesion created a lumen-within-lumen configuration. Careful inspection allowed the identification of the true duodenal lumen adjacent to the diverticular sac ( Fig. 2 ).

Endoscopic identification of an intraluminal duodenal diverticulum showing a characteristic double-lumen appearance and the true duodenal lumen before planned ERCP. ERCP, endoscopic retrograde cholangiopancreatography.Video 1

Recognition of this uncommon anomaly before ERCP is important because IDD may obscure or displace the papillary region and complicate cannulation 2 3 4 . Awareness of its characteristic endoscopic appearance helps avoid misinterpretation as a subepithelial lesion and facilitates appropriate therapeutic planning.

Endoscopy_UCTN_Code_CCL_1AB_2A
